# Reply: Did animal offer relevant model for bevacizumab testing?

**DOI:** 10.1038/sj.bjc.6604694

**Published:** 2008-10-07

**Authors:** A Bozec, A Sudaka, J L Fischel, M C Brunstein, M C Grimaldi, G Milano

**Affiliations:** 1 Centre Antoine Lacassagne, Oncopharmacology Unit, 33 Avenue de Valombrose, Nice Cedex 2 0 6189, France; 2 Pathology Laboratory, Centre Antoine Lacassagne, 33 Avenue de Valombrose, Nice Cedex 2 06189, France

**Sir,**

We read with great attention the letter by Eveno *et al*, concerning our article TH/2008/4030R published in *Br J Cancer* 99/1 July 2008.

The main point of their letter is that murine models are not suited as evidence of an effect of bevacizumab on tumour growth, because bevacizumab fails to efficiently neutralise murine VEGF.

Their experiments have been performed *in vitro* on isolated endothelial cells, whereas ours have been performed in xenografted animals. In our conditions human VEGF, produced in very high quantities by the CAL33 human cancer cells, certainly outnumbered the murine VEGF at least inside the tumour and in its immediate environment. It is not excluded that a greater dose of bevacizumab could perhaps have been more efficient.

Anyway, the purpose of our experiments was not to test the effect of bevacizumab alone on CAL33 growth but rather its interaction with erlotinib and radiation.

Another explanation of the lack of efficiency of bevacizumab given alone could result from the orthotopic model used with a very short time given to the drug to exhibit its effects (10 days after tumour cells injection, 7 days after the beginning of treatment) giving treatment on small tumours at a moment when tumour-driven angiogenesis is not particularly determinant. What gives credit to this explanation are the results of another recent study of our group (Bozec *et al*, 2008) in which we tested bevacizumab, AZD2171, a VEGFR tki, and their combination on CAL33 cells growing as a classical xenograft in the flank of animals. The treatment this time started when tumour volume reached 250 mm^3^, 12 days after tumour cell injection (i.e., 2 days after tumour collection in the *Br J Cancer* article) and the effect of bevacizumab was clearly evident 4 days after the beginning of treatment and lasted until the end of the observation period, 26 days after the beginning of treatment.

Moreover, many earlier preclinical studies performed on xenografted tumours (for review see Gerber and Ferrara, 2005) demonstrated an effect of bevacizumab on tumour growth.

We agree with Eveno *et al* that the present animal models are far from being a perfect representation of the clinical situation and that animals genetically modified to secrete human VEGF in place of murine VEGF model could be an improvement to the present situation.
